# Modeling the Potential Distribution Patterns of the Invasive Plant Species *Phytolacca americana* in China in Response to Climate Change

**DOI:** 10.3390/plants13081082

**Published:** 2024-04-12

**Authors:** Qianru Nan, Chunhui Li, Xinghao Li, Danni Zheng, Zhaohua Li, Liya Zhao

**Affiliations:** 1 School of Resources and Environmental Science, Hubei University, Wuhan 430062, China; 202121108012639@stu.hubu.edu.cn (Q.N.); li_xhao@163.com (X.L.); 202221108012260@stu.hubu.edu.cn (D.Z.); zli@hubu.edu.cn (Z.L.); 2 Agricultural Development Service Centre of Enshi Tujia and Miao Autonomous Prefecture, Enshi 44500, China

**Keywords:** *Phytolacca americana*, biological invasion, Maxent, climate change, potentially suitable area

## Abstract

*Phytolacca americana*, introduced to China in the 20th century for its medicinal properties, has posed a significant ecological and agricultural challenge. Its prolific fruit production, high reproductive coefficient, adaptability, and toxic roots and fruits have led to the formation of monoculture communities, reducing native species diversity and posing threats to agriculture, human and animal health, and local ecosystems. Understanding its potential distribution patterns at a regional scale and its response to climate change is essential for effective monitoring, management, and control. In this study, we utilized the Maxent model to simulate potential habitat areas of *P. americana* across three timeframes (current, 2050s, and 2070s) under three climate change scenarios (SSP126, SSP245, and SSP585). Leveraging data from 556 *P. americana* sites across China, we employed ROC curves to assess the prediction accuracy. Our findings highlight key environmental factors influencing *P. americana*’s geographical distribution, including the driest month’s precipitation, the coldest month’s minimum temperature, the wettest month’s precipitation, isothermality, and temperature annual range. Under current climate conditions, *P. americana* potentially inhabits 280.26 × 10^4^ km^2^ in China, with a concentration in 27 provinces and cities within the Yangtze River basin and its southern regions. While future climate change scenarios do not drastically alter the total suitable area, the proportions of high and low-suitability areas decrease over time, shifting towards moderate suitability. Specifically, in the SSP126 scenario, the centroid of the predicted suitable area shifts northeastward and then southwestward. In contrast, in the SSP245 and SSP585 scenarios, the centroid shifts northward.

## 1. Introduction

Recently, intensified global environmental changes have significantly increased the threat of biological invasions to ecosystems and biodiversity [1]. Given the backdrop of significant global climate change, understanding the spatial distribution of biological populations and their interplay with the environment has become pivotal for unraveling biodiversity patterns and devising strategies for its preservation [2]. Extensive research demonstrates that climate change profoundly impacts and will continue to influence species distribution patterns [3,4]. Consequently, predicting species’ potential habitats and their migration trends in response to climate change is vital for informing future species management, ecological conservation, and cultivation strategies [5,6].

The emergence of advanced computational statistical techniques and the development of global information systems have facilitated the exploration of direct correlations between environmental factors, such as climate and topography, and species data, revolutionizing the field of ecology [7,8]. Among these innovations, species distribution models (SDMs) have emerged as a crucial tool for deciphering species distribution patterns [9]. SDMs leverage known distribution data and corresponding environmental variables to simulate species’ geographical distribution and their responses to climate change, employing various algorithms across temporal and spatial scales [2,10].

The Maxent model, based on the maximum entropy principle developed by Phillips, is widely recognized for predicting geographical distributions of species [11,12,13,14]. Its notable advantage lies in its capacity to maintain a high accuracy and stability, even in cases with incomplete species data and limited sample sizes [15]. Furthermore, Maxent stands out for its speedy computations and flexible operation [14,15,16], with its predictions easily visualized using ArcGIS [17]. The Maxent model has also proven valuable in forecasting the potential distribution of invasive and endangered species, including *Xanthium spinosum* L. (Asteraceae) [18], *Lonicera japonica* Thunb. (Caprifoliaceae) [19], *Ziziphus jujuba* var. *spinosa* (Rhamnaceae) [20], *Pinus massoniana* Lamb. (Pinaceae) [21], and *Litsea cubeba* (Lour.) Pers. (Lauraceae) [22].

Native to North America, *Phytolacca americana*, a perennial herb from the Phytolaccaceae family, was introduced to China for its medicinal properties. Since its discovery in Hangzhou in 1935, it has rapidly invaded numerous regions of the country, colonizing forest margins, roadsides, village lands, wastelands, and disrupting tea plantations, orchards, and bamboo groves [23]. This invasion poses significant threats to local agricultural and forestry production and biodiversity [24,25]. *P. americana* was included in the “List of Invasive Species in China’s Natural Ecosystems (Fourth Batch)” in 2016, mandating strict control and regulation of its cultivation [26]. Consequently, conducting a comprehensive study of *P. americana*’s suitable and potential distribution areas assumes a paramount importance for the effective monitoring, early warning, and prevention of its invasion. It is also critical for sustaining agroforestry production, conserving diversity, maintaining productivity, and restoring ecological environments. Presently, research on the invasive species *P. americana* primarily focuses on its invasion mechanisms, biological characteristics, and ecological hazards [27,28,29]. However, there is limited domestic and international research concerning its suitable distribution areas and potential sites.

In this study, leveraging distribution data and relevant environmental variables, we employed the Maxent model and ArcGIS to forecast potential suitable areas for *P. americana* across various climate scenarios, both current and future. Our objectives encompassed (1) predicting potentially suitable habitats for *P. americana* under current climate scenarios, (2) identifying primary environmental variables constraining its geographic distribution, and (3) forecasting potentially suitable habitats under future climate scenarios. Our aim was to unravel shifts in *P. americana*’s present and future geographical distribution patterns. This research strives to provide a scientific foundation for the monitoring and management of this highly invasive species.

## 2. Materials and Methods

### 2.1. Collection and Screening of Species Occurrence Data

The distribution data for *P. americana* in China were primarily sourced from two databases: the Global Biodiversity Information Facility (GBIF) (https://www.gbif.org, accessed on 1 May 2023) and the Chinese National Plant Specimen Resource Center (http://www.cvh.ac.cn/, accessed on 1 May 2023). Additionally, we augmented our dataset with field survey data collected using GPS terminals. The data collection period spanned from 1935 to 2023.

**Figure 1:**
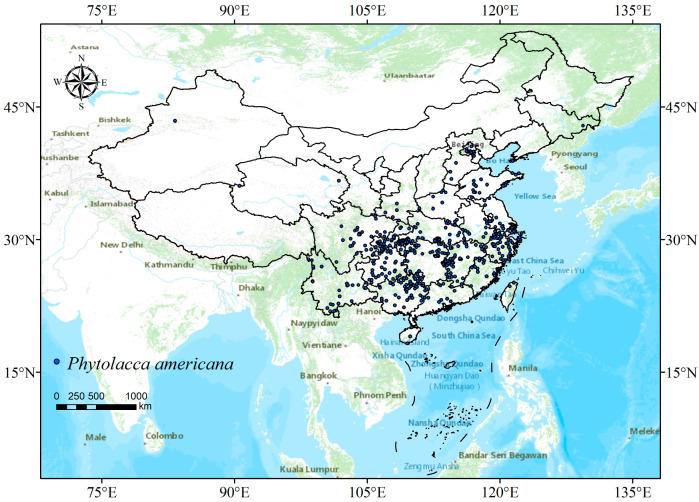
Current distribution of *Phytolacca americana* L. across China, based on data compiled up to May 2023.

To ensure data accuracy, we performed a thorough screening of the occurrence data, eliminating duplicate entries and addressing missing latitude and longitude information. This meticulous data curation process yielded a comprehensive dataset consisting of 556 unique distribution points for *P. americana* across China. The resulting distribution map is depicted in Figure 1.

### 2.2. Data Acquisition and Variables

We sourced historical and projected climate data from the World Climate Database (https://www.worldclim.org, accessed on 1 May 2023), with a spatial resolution of 2.5 arc minutes (approximately 5 km × 5 km). The selected periods for analysis encompassed current climate conditions (1970–2000), as well as two future projections, the 2050s (2041–2060) and the 2070s (2061–2080), as outlined in Table 1.

To model the future scenarios, we utilized data from the Beijing Climate Center (BCC) Climate System Model 2 Medium Resolution, within the framework of the Sixth International Coupled Model Intercomparison Program (CMIP6). This dataset included projections corresponding to three distinct Shared Socioeconomic Pathways (SSPs): SSP1_2.6 (sustainable development), SSP2_4.5 (moderate development), and SSP5_8.5 (normal development) [30].

### 2.3. Variable Selection

To mitigate the impact of spatial covariance among environmental variables on the accuracy of the Maxent model and enhance its predictive performance, we employed a systematic variable selection process guided by the following principles:

A Maxent pre-model was constructed using 20 environmental variables, allocating 25% of the data for testing and 75% for training. Default parameters were utilized, and the calculations were repeated ten times with the Bootstrap method. A total of 500 iterations and 10,000 background points were specified. We employed the jackknife test to assess the contribution of each environmental variable [30,31,32,33].

A Principal Component Analysis (PCA) was conducted, using SPSS 25.0 software, on the initial set of 20 environmental variables. Principal components with eigenvalues exceeding 1 were retained. From each principal component, environmental variables with loading coefficients exceeding 0.5 were selected for further analysis. Spearman correlation tables were generated [34].

Exclusion Criteria: We excluded environmental variables with a high correlation (|r| ≥ 0.8), low percentage contribution, and limited biological significance [35]. When two environmental variables exhibited a correlation coefficient exceeding 0.8, the less influential variable was eliminated using the jackknife test in Maxent. Only variables with greater contributions to the model were retained [36,37]. Ultimately, we identified nine environmental variables for the Maxent modeling process: bio2, bio3, bio6, bio7, bio8, bio10, bio12, bio13, and bio14.

### 2.4. Parameter Selection and Model Accuracy Evaluation

We employed Maxent 3.4.1, following the methodology proposed by Phillips et al. (2017) [38], to construct the maximum entropy model for our study. To ensure the probability of *P. americana* distribution approached normality, we allocated 75% of the data for model training, reserving the remaining 25% for accuracy validation [39]. The primary parameters were configured as follows: Maximum Iterations: 500, Replicated Run Type: Bootstrap, No. of Replicates: 10, and Output Format: logistic.

We incorporated *P. americana* distribution data and nine environmental variables into the Maxent model. The “Do jackknife” function within the software was employed to assess the contribution of each environmental factor to the model construction [40,41]. Response curves were generated to visualize the relationship between distribution probabilities and climate factors (see Figure S1). The model accuracy was evaluated using the receiver operating characteristic (ROC) curve [17]. We conducted 10 model replicates, and the average result was considered the prediction of potential species distribution [42].

The model performance was assessed using the ROC area under the curve (AUC) [43]. The AUC is independent of the model threshold settings and serves as a reliable measure of accuracy [43]. AUC values range from 0 to 1, with interpretations as follows: an AUC < 0.7 indicates a poor predictive performance, from 0.7 to 0.8 suggests moderate performance, from 0.8 to 0.9 signifies good performance, and from 0.9 to 1.0 indicates excellent performance [44]). Upon running the model with the specified parameter settings in ten replicates, the average AUC from these iterations was calculated and found to be 0.907 (see Figure S2). This high AUC value indicates an excellent predictive performance, underscoring the accuracy and credibility of our prediction results.

### 2.5. Classification of Suitable Areas

We took the output ASC file from Maxent and imported it into ArcGIS, converting it into grid data. It was then overlaid onto a map of China’s administrative districts for visualization. Using ArcGIS, we employed the reclassification function to classify different areas and utilized grid calculation tools to determine their corresponding areas. For this study, we employed the Natural Breaks (Jenks) classification method to categorize suitable areas into four grades: highly suitable areas (*p* ≥ 0.48), moderately suitable areas (0.30 ≤ *p* < 0.48), low-suitability areas (0.10 ≤ *p* < 0.30), and unsuitable areas (*p* < 0.10).

## 3. Results

### 3.1. Analysis of the Contribution of Environmental Variables

Following model optimization, the final model included nine bioclimatic factors:

Mean diurnal range (bio2), isothermality (bio3), minimum temperature of coldest month (bio6), temperature annual range (bio7), mean temperature of wettest quarter (bio8), mean temperature of warmest quarter (bio10), annual precipitation (bio12), precipitation of wettest month (bio13), precipitation of driest month (bio14). Their respective contributions to the model construction are as follows: bio14: 68.7%, bio6: 13.6%, bio13: 6.7%, bio3: 3.4%, bio7: 2.3%, bio12: 1.7%, bio2: 1.6%, bio8: 1.1%, and bio10: 0.9% (Table 2).

### 3.2. Current Potentially Suitable Areas of P. americana in China

**Figure 2:**
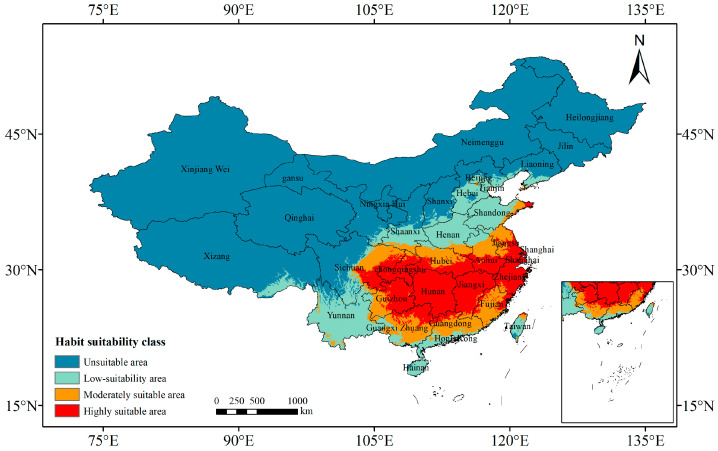
Predictions of the potentially suitable area of *Phytolacca americana* L. under current climate conditions based on the Maxent model. The color-coded areas denote different levels of suitability: red, highly suitable areas; orange, moderately suitable areas; light blue, low-suitability areas; and blue, unsuitable areas.

Figure 2 illustrates the division of *P. americana* distribution based on the Maxent model predictions, visualizing its potential suitable areas. Under current climate conditions, potentially suitable areas for *P. americana* are dispersed across 27 provinces and municipalities, including the Hong Kong and Macao Special Administrative Regions. These areas are predominantly concentrated in the Yangtze River basin and its southern vicinity.

Highly suitable areas are primarily situated in southeastern Sichuan, eastern Guizhou, southern Hubei, southern Anhui, southern Jiangsu, northern Guangdong, the northern Guangxi Zhuang Autonomous Region, northwestern and southeastern Fujian, Zhejiang, Shanghai, Jiangxi, Hunan, and Chongqing, with a sporadic presence in eastern Shandong and northern Taiwan. These highly suitable areas cover a total area of 98.68 × 10^4^ km^2^, constituting 10.26% of China’s total area.

Moderately suitable areas border the highly suitable regions, encompassing a total area of 69.10 × 10^4^ km^2^, accounting for 24.66% of the total suitable area.

Low-suitability areas predominantly include northeastern Sichuan, western Guizhou, central Hubei, central Anhui, northern Jiangsu, central Guangdong, the central Guangxi Zhuang Autonomous Region, most of Fujian, southern Henan, and southern Shaanxi, covering a total area of 112.48 × 10^4^ km^2^, which is 40.13% of the total suitable area.

The combined area of low-suitability and unsuitable areas reaches 793.99 × 10^4^ km^2^, representing 82.55% of China’s total area.

### 3.3. Future Suitable Areas of P. americana under Different Climate Scenarios

**Figure 3:**
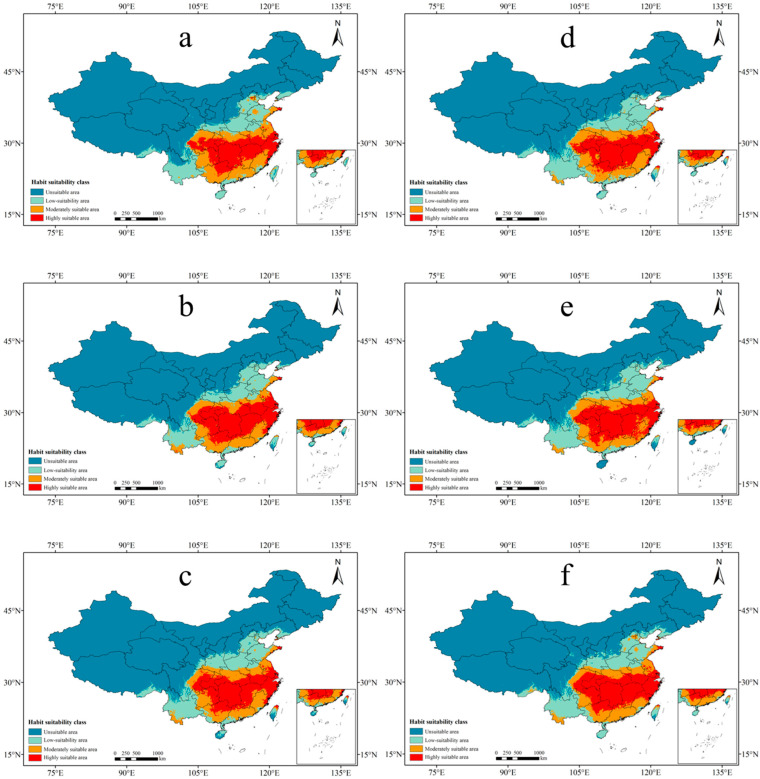
Dynamic change map of the predicted potentially suitable area of *Phytolacca americana* L. based on different climate scenarios in the future. ((**a**) 2050s-SSP126; (**b**) 2050s-SSP245; (**c**) 2050s-SSP585; (**d**) 2070s-SSP126; (**e**) 2070s-SSP245; (**f**) 2070s-SSP585).

**Figure 4:**
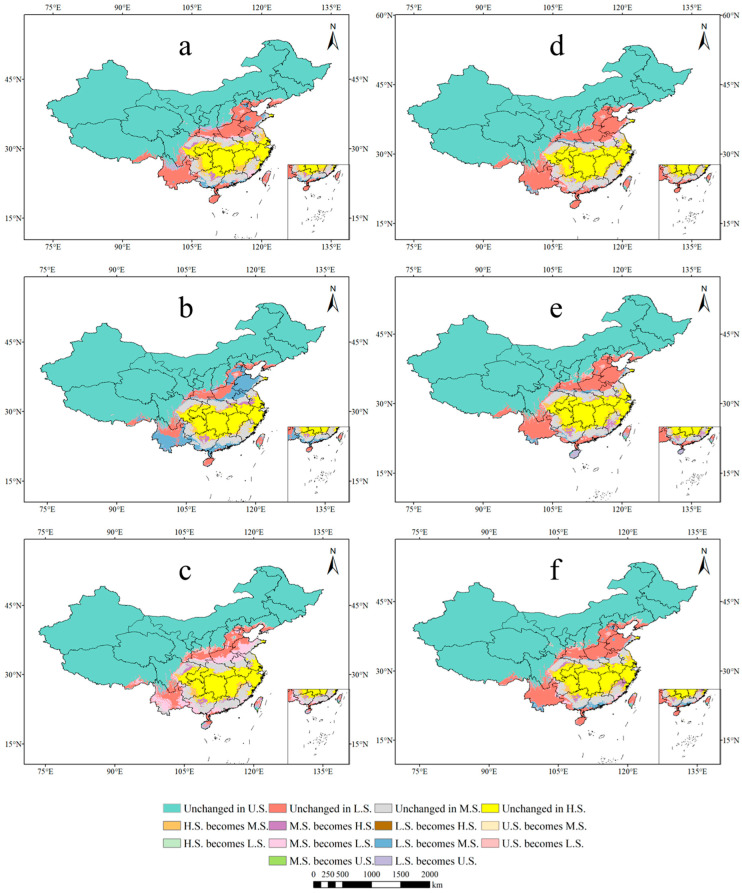
Predictions of the potentially suitable area of *Phytolacca americana* L. under different future climate scenarios based on the Maxent model. ((**a**) 2050s-SSP126; (**b**) 2050s-SSP245; (**c**) 2050s-SSP585; (**d**) 2070s-SSP126; (**e**) 2070s-SSP245; (**f**) 2070s-SSP585; U.S.: unsuitable; L.S.: low-suitability; M.S.: moderately suitable; H.S.: highly suitable).

We employed the Maxent model, following the same criteria as mentioned earlier, to predict the potential suitability areas for *P. americana* in the 2050s and 2070s under three climate scenarios: SSP126, SSP245, and SSP585. The results included a spatial distribution map of future predicted potential suitability areas for *P. americana* (Figure 3), a dynamic change map of each suitability class (Figure 4), and changes in area for each suitability category (Table 3).

Across the three climate scenarios in the 2050s and 2070s, the location and area of each suitability class for *P. americana* exhibited varying degrees of change (Table 3). Notably, there was a significant increase in moderately suitable areas and an overall decrease in high- and low-suitability areas. There was a modest increase in highly suitable areas in the 2050s under the SSP245 and SSP585 scenarios and in low-suitability areas in the 2070s under the SSP126 scenario. The percentage change in the total area of suitable areas increased over time, with varying trends under different climate scenarios.

Looking specifically at moderately suitable areas, they increased in size over time under all three climate scenarios, with reduced fragmentation (Figure 3 and Figure 4). In the 2050s, the SSP126 scenario exhibited the largest area of moderately suitable regions (82.6 × 10^4^ km^2^), marking a 19.53% increase compared to current climatic conditions, representing the most significant change (Table 3). The expansion of moderately suitable areas was primarily attributed to transformations from highly suitable areas at multiple junctions and from low-suitability areas in various regions. In the 2070s, the SSP126 scenario continued to lead, with the largest moderately suitable area (76.09 × 10^4^ km^2^), reflecting a 10.10% increase (Table 3).

In contrast, the total area of highly suitable and low-suitability areas declined, primarily converting into moderately suitable areas. The most substantial decrease in highly suitable areas occurred in the 2050s under the SSP126 scenario, with a 15.86% reduction (Table 3). The most significant decline in low-suitability areas was observed in the 2050s under the SSP245 scenario, with a 9.14% decrease (Table 3). Many of the areas transitioning from low-suitability to moderately suitable became unsuitable, notably in northern Yunnan, southern Liaoning, and the junction of Shaanxi, Shanxi, and Henan (Figure 4).

### 3.4. Centroid Distributional Shifts under Future Climate Conditions

**Figure 5:**
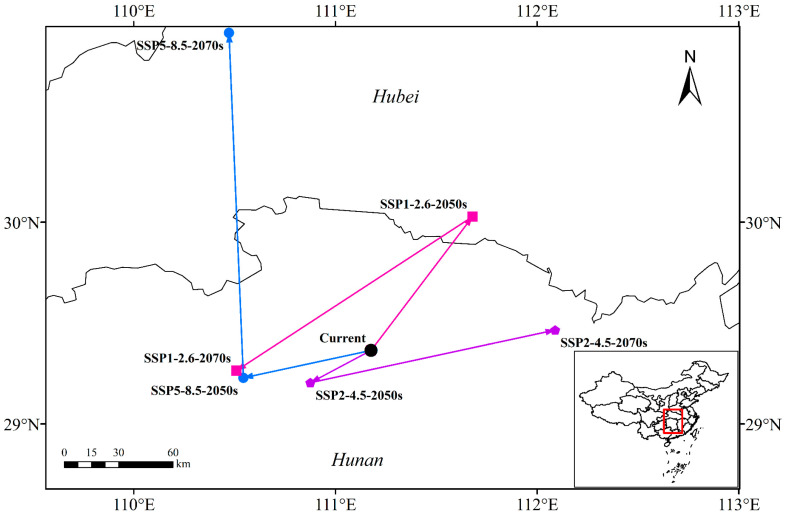
Total suitable habitat centroid distribution shifts under climate change for *Phytolacca americana* L. (The red square represents the location in China of the main migration areas of the centroids.).

Figure 5 illustrates the calculation of the centroid location, direction, and distance using ArcGIS under various climate scenarios and time periods.

Under current climate conditions, the centroid of suitable habitat was at coordinates 111°10′36″ E, 29°21′51″ N.

For the SSP126 climate scenario:

In the 2050s, the centroid was projected to shift 88.54 km to the northeast (111°40′47″ E, 30°1′42″ N). In the 2070s, it was expected to move 142.04 km to the southwest (111°30′32″ E, 29°15′51″ N). Overall, it would have shifted 66.09 km southwest.

For the SSP245 climate scenario:

In the 2050s, the centroid was projected to move 34.33 km southwest (110°52′33″ E, 29°12′15″ N). In the 2070s, it was expected to shift 121.75 km northeast (112°5′22″ E, 29°27′54″ N). Overall, it would have moved 89.54 km to the northeast.

For the SSP585 climate scenario:

In the 2050s, the centroid was projected to move 63.59 km southwest (110°32′37″ E, 29°13′42″ N). In the 2070s, it was expected to shift 190.61 km north (110°28′19″ E, 30°56′21″ N). Overall, it would have moved 188.08 km to the northwest.

## 4. Discussion

### 4.1. Main Environmental Variables Affecting the Occurrence of P. americana

The Maxent model, considering percentage contribution, ranked importance, and jackknife tests, identifies the primary factors influencing the distribution of *P. americana*. Precipitation of the driest month, minimum temperature of the coldest month, precipitation of the wettest month, isothermality, and temperature annual range emerge as the dominant variables shaping its current distribution.

These findings highlight the pivotal roles of temperature and precipitation in determining the species’ distribution. Research has shown that *P. americana* thrives in warm and humid climates [45]. Temperature affects plant photosynthesis, while precipitation plays a critical role in regulating carbon sequestration and transpiration by influencing soil moisture. Together, these factors impact various physiological processes throughout the plant’s life cycle, from seed germination to growth and development [46]. Hydrothermal conditions are key variables that restrict the geographical distribution of plants. Terrestrial plants exhibit high sensitivity to temperature and precipitation [47,48]. Consequently, areas with warm and humid climatic conditions are more conducive to the growth and proliferation of *P. americana*. These environmental insights provide valuable information for understanding the current distribution patterns of *P. americana* and can be instrumental in managing and controlling its spread.

The present study reveals that *P. americana* primarily inhabits coastal regions rich in hydrothermal resources and the subtropical monsoon climate zone south of the Yangtze River in China. Several factors contribute to this distribution pattern.

Climatic Suitability: The species thrives in areas characterized by humid and rainy summers, seasonal high temperatures, and ample sunshine—conditions conducive to its growth and reproduction [49,50]. These climatic characteristics align with the observed distribution.

Human Activities: Human interventions, including agricultural production, trade, and transportation, often serve as significant drivers in the spread of invasive species like *P. americana* [51,52]. Frequent economic and trade exchanges in the distribution areas may contribute to its expansion.

Unsuitable Regions: Conversely, regions such as the Tibetan Plateau, inland Northwest China, and much of Northeast China are unsuitable for *P. americana*. These areas feature temperate continental and temperate monsoon climates, characterized by low annual rainfall and cold, dry winters. As such, they pose a low risk of invasion or are entirely unsuitable for the species’ growth.

In summary, the current distribution of *P. americana* can be attributed to a combination of favorable climatic conditions, human activities, and the unsuitability of certain regions. These insights are valuable for understanding the species’ distribution patterns and can inform management and control efforts.

### 4.2. Future Changes in P. americana Distribution

The study indicates that the potential suitable areas for *P. americana* will undergo changes over time and under various climate scenarios. While specific patterns may vary, a consistent trend emerges: no significant alteration in the total suitable area, expansion of moderately suitable areas, and reductions in highly suitable and low-suitability areas.

Climate-Induced Shifts in Centroid Location:

Depending on future climatic conditions, the centroid of *P. americana* suitability exhibits directional movement. Under the SSP245 and SSP585 scenarios, it generally migrates to higher latitudes over time. SSP585 shows more pronounced shifts, especially by the 2070s. Conversely, under the SSP126 scenario, the centroid moves northeastward in the 2050s but significantly shifts southwestward by the 2070s.

Implications of Climate Change:

Centroid movements in the present study (Figure 5) align with previous findings that climate change can drive shifts in species distributions. Examples include *P. massoniana* extending northward [21], *Vaccinium membranaceum* Douglas ex Torr. (Ericaceae) transitioning to high-elevation areas [53], and *Z. spinosa*’s suitable area gradually moving northward [20].

Factors Influencing Shifts:

The SSP126 scenario, projecting reduced carbon dioxide emissions and temperature stabilization, leads to a relatively slow rate of temperature increase. Consequently, *P. americana* suitability does not exhibit a significant northward shift, reflecting the influence of sustainable development and a slower temperature rise.

**Figure 6:**
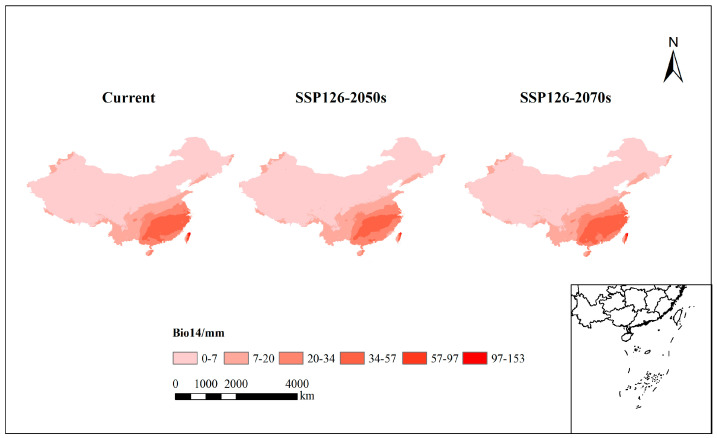
Changes in the highest-contributing climate variable, precipitation of the driest month (bio14), under the SSP126 future climate scenario versus the current climate scenario for *Phytolacca americana* L.

Hydrothermal Considerations:

The subtropical monsoon climate of high-suitability areas plays a crucial role. Changes in precipitation patterns under the SSP126 scenario significantly impact suitability. Precipitation of the driest month (bio14), a critical contributing factor, tends to fluctuate, influencing the centroid’s movement (Figure 6).

In summary, the study highlights the potential impact of climate scenarios on *P. americana* distribution. The findings underscore the importance of climate-induced shifts in species habitats and the role of precipitation patterns in shaping these changes.

### 4.3. Prevention and Control Strategies

China’s location in the East Asian monsoon zone, with its proximity to the Pacific Ocean, exposes it to significant climate change impacts. Studies indicate that rising temperatures, strengthened summer winds, increased rainfall, and a warmer, wetter climate are expected in coastal areas due to global climate change. These changes align with *P. americana*’s preference for warm and humid conditions, potentially expanding its suitable habitat in the future [54,55,56].

The modeled potential distribution maps in this study serve as crucial references for effective prevention and control strategies for *P. americana*. Three key recommendations are proposed:

Strict Regulation of Highly Suitable Areas: Regions such as Zhejiang, Shanghai, Jiangxi, Hunan, and Chongqing are identified as highly suitable for *P. americana* under the current climate scenario. Consequently, strict regulations should be enforced to control the introduction and planting of this invasive species in these areas.

Enhanced Monitoring for New Suitable Habitats: Proactive monitoring of potential new suitable habitats should be intensified to prevent *P. americana* invasion driven by human activities. Identifying and addressing emerging suitable areas can help curb its spread.

Protection of Agricultural Areas: Agricultural production zones, including croplands and orchards, require robust prevention and control measures against *P. americana* intrusion to safeguard native species. Early intervention methods, such as chemical and biological controls, as well as the timely removal of fruit-bearing plants and aerial parts after fruiting, can effectively mitigate its impact.

These recommendations underscore the importance of proactive and adaptive management strategies to address the potential expansion of *P. americana* in response to climate change, safeguarding native ecosystems and agricultural productivity.

### 4.4. Limitations of the Study

While our study provides valuable insights into the potential distribution of *P. americana*, several limitations should be acknowledged:

Limited Environmental Factors: Our analysis focused solely on bioclimatic variables as predictors of species distribution. Species distributions are influenced by a myriad of biotic factors (e.g., interspecific competition, predation, and disease) and abiotic factors (e.g., soil, topography, and human activities). Future studies should consider a broader range of factors, such as soil type, land use, and anthropogenic impacts, to provide a more comprehensive understanding of *P. americana*’s distribution dynamics.

Incomplete Response Curve Data: Although we provided response curves for dominant environmental factors, some secondary factors, such as elevation, annual mean temperature, and temperature seasonality, were not included due to data limitations. Incorporating these factors into future research could enhance the precision of our models.

Limited Geographic Scope: Our dataset only included occurrences within China; it does not fully capture the global distribution of *P. americana*. The response curves derived from this dataset may not entirely represent the species’ environmental responses across its entire range. Future studies should aim to gather data from a broader geographic range to improve model accuracy.

Large-Scale Analysis: Our study primarily operates at a large geographic scale. Future research could benefit from conducting more fine-scale analyses in specific geographic regions. Excluding unsurveyed and inaccessible areas in these smaller-scale studies can enhance precision and accuracy.

Model Validation: While the Maxent model is widely accepted for species distribution modeling, employing alternative models, such as BioCLIM and DOMAIN, in future research could provide additional validation and strengthen our findings.

Acknowledging these limitations helps refine the scope of future research and ensures a more comprehensive understanding of *P. americana*’s distribution patterns and ecological interactions.

## 5. Conclusions

In this study, we employed the Maxent model to predict the potential suitable areas for *P. americana* in China. Examining future climate scenarios, our projections indicate that the total area of potential suitable habitat for *P. americana* is unlikely to undergo significant changes. However, the centroid of suitable areas is expected to shift northeastward in the 2050s under the SSP126 climate scenario, followed by a substantial southwestward movement in the 2070s. Under the SSP245 and SSP585 climate scenarios, the centroid generally migrates toward higher latitudes over time.

Considering these findings, we propose rigorous monitoring of the currently highly suitable areas for *P. americana*, especially agricultural production areas such as croplands and gardens. Meanwhile, we recommend intensified surveillance of potential new suitable habitats, based on projected future distribution patterns of the species. Additionally, to address the limitations of this study, future research can further improve the results of this study by considering a broader range of environmental factors (e.g., soil type, land use, and anthropogenic impacts), a wider range of geographic factors (e.g., global scale), and more model validation (e.g., BioCLIM, DOMAIN, etc.). In conclusion, the outcomes of this study offer crucial insights for future monitoring, early warning systems, and management strategies concerning *P. americana*.

## Figures and Tables

**Table 1:** Environmental variables considered for modeling the potentially suitable habitat of *Phytolacca americana* L.

Environmental Variables	Names
2.5m-bio1	Annual mean temperature
2.5m-bio2	Mean diurnal range
2.5m-bio3	Isothermality (2/7) (×100)
2.5m-bio4	Temperature seasonality (standard deviation × 100)
2.5m-bio5	Max temperature of warmest month
2.5m-bio6	Min temperature of coldest month
2.5m-bio7	Temperature annual range
2.5m-bio8	Mean temperature of wettest quarter
2.5m-bio9	Mean temperature of driest quarter
2.5m-bio10	Mean temperature of warmest quarter
2.5m-bio11	Mean temperature of coldest quarter
2.5m-bio12	Annual precipitation
2.5m-bio13	Precipitation of wettest month
2.5m-bio14	Precipitation of driest month
2.5m-bio15	Precipitation seasonality (variation coefficient)
2.5m-bio16	Precipitation of wettest quarter
2.5m-bio17	Precipitation of driest quarter
2.5m-bio18	Precipitation of warmest quarter
2.5m-bio19	Precipitation of coldest quarter
Alt	Altitude

**Table 2:** Contribution rate of environmental variables.

Variable	Bioclimatic Factors	Percent Contribution
bio14	Precipitation of driest month	68.7
bio6	Min temperature of coldest month	13.6
bio13	Precipitation of wettest month	6.7
bio3	Isothermality (2/7) (×100)	3.4
bio7	Temperature annual range	2.3
bio12	Annual precipitation	1.7
bio2	Mean diurnal range	1.6
bio8	Mean temperature of wettest quarter	1.1
bio10	Mean temperature of warmest quarter	0.9

**Table 3:** Potential suitable area of *Phytolacca americana* L. under different climate change scenarios.

Scenario	Period	Highly Suitable Area	Change	Moderately Suitable Area	Change	Low-Suitability Area	Change	Total Area	Total Change
		Area (×10^4^ km^2^)		Area (×10^4^ km^2^)		Area (×10^4^ km^2^)			
Current		98.68		69.10		112.48		280.26	
SSP126	2050s	83.02	−15.86%	82.60	19.53%	104.94	−6.70%	270.56	−3.46%
	2070s	87.51	−11.32%	76.09	10.10%	115.99	3.12%	279.59	−0.24%
SSP245	2050s	101.00	2.35%	70.19	1.58%	102.20	−9.14%	273.40	−2.45%
	2070s	97.46	−1.24%	72.50	4.91%	107.49	−4.43%	277.45	−1.00%
SSP585	2050s	94.42	−4.32%	79.07	14.43%	111.99	−0.44%	285.48	1.86%
	2070s	102.50	3.88%	74.93	8.42%	109.01	−3.08%	286.44	2.20%

## Data Availability

Data are contained within the article and Supplementary Materials.

## Supplementary Materials

The following supporting information can be downloaded at: https://www.mdpi.com/article/10.3390/plants13081082/s1, Figure S1: Response curve of environmental factors. Figure S2: Reliability test of the distribution model created for *Phytolacca americana* L.
