# Supplementary material for: Modeling the Potential Distribution Patterns of the Invasive Plant Species Phytolacca americana in China in Response to Climate Change
Source: Plants (Basel). 2024 Apr 12;13(8):1082. doi: 10.3390/plants13081082 (PMC11054219; doi:10.3390/plants13081082)
Supplement: Supplementary file 1 [file plants-13-01082-s001.zip › plants-2929116-supplementary.pdf]

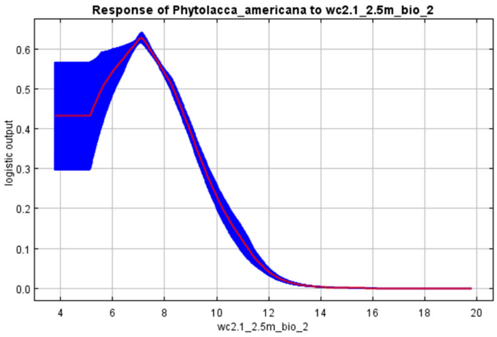
Response of Phytolacca_americana to wc2.1_2.5m_bio_2
logistic output
wc2.1_2.5m_bio_2

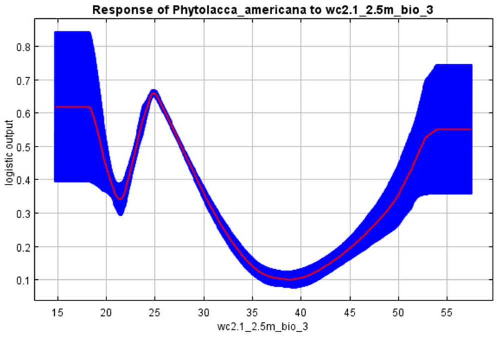
Response of Phytolacca_americana to wc2.1_2.5m_bio_3
logistic output
wc2.1_2.5m_bio_3

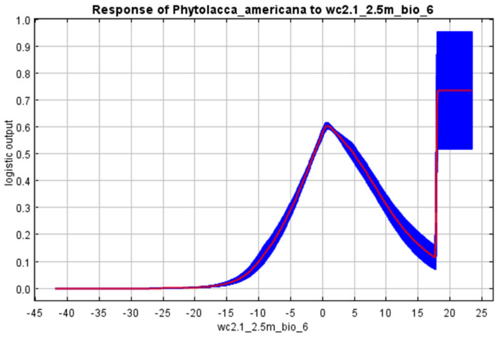
Response of Phytolacca_americana to wc2.1_2.5m_bio_6
logistic output
wc2.1_2.5m_bio_6

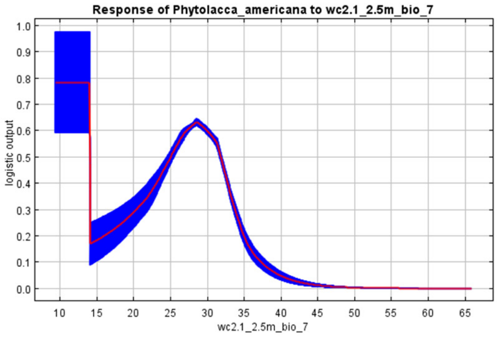
Response of Phytolacca_americana to wc2.1_2.5m_bio_7
logistic output
wc2.1_2.5m_bio_7

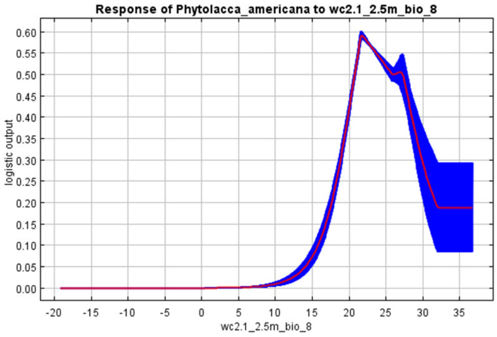
Response of Phytolacca_americana to wc2.1_2.5m_bio_8
logistic output
wc2.1_2.5m_bio_8

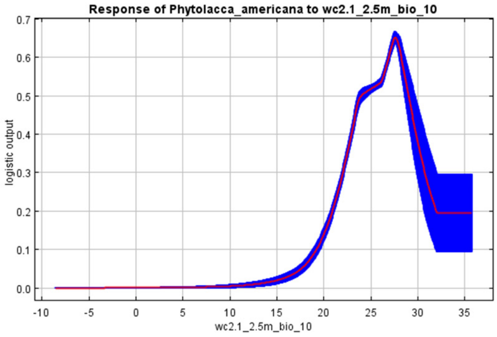
Response of Phytolacca_americana to wc2.1_2.5m_bio_10
logistic output
wc2.1_2.5m_bio_10

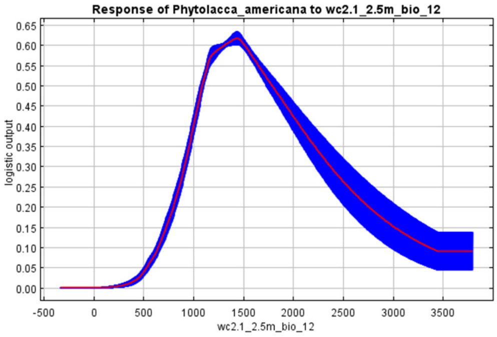
Response of Phytolacca_americana to wc2.1_2.5m_bio_12
logistic output
wc2.1_2.5m_bio_12

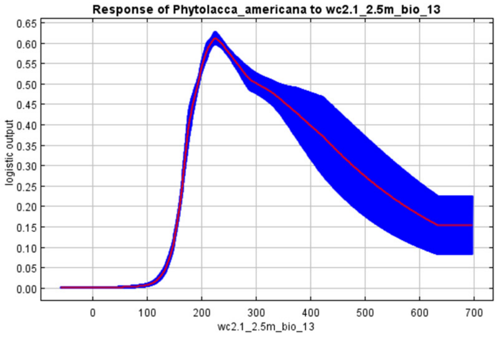
Response of Phytolacca_americana to wc2.1_2.5m_bio_13
logistic output
wc2.1_2.5m_bio_13

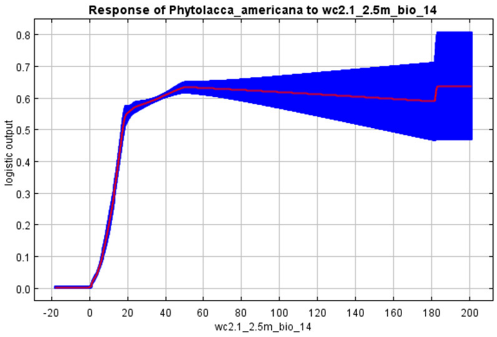

Supplementary Figure S1: Response curve of environmental factors.

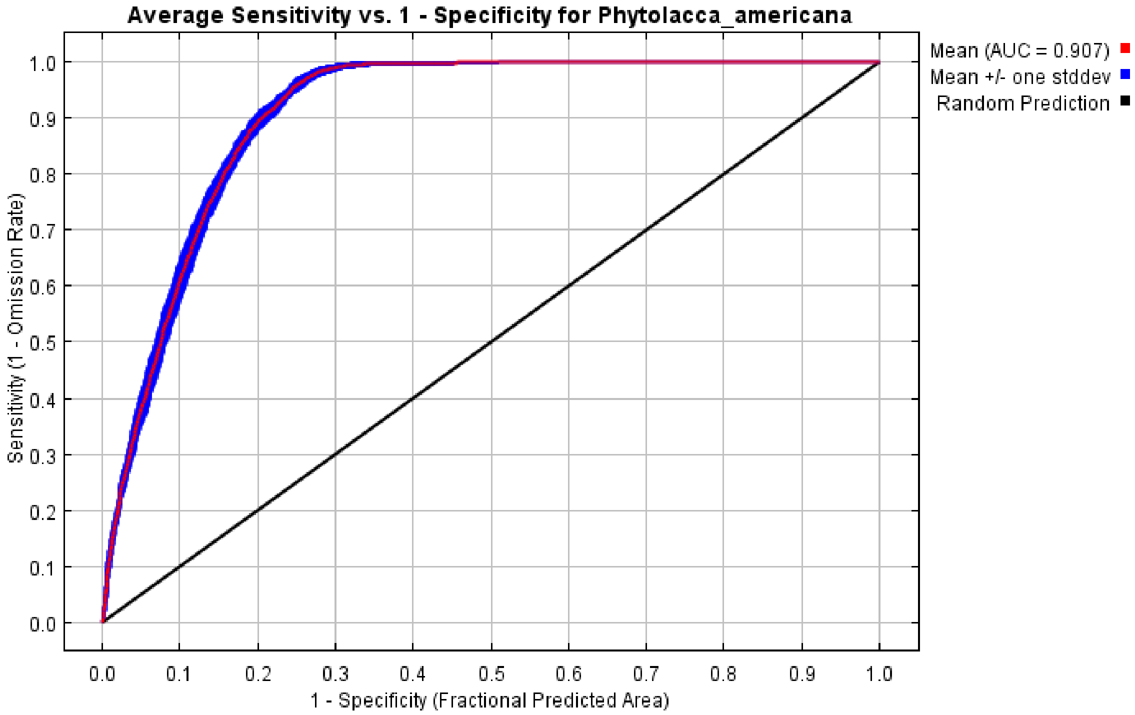

Supplementary Figure S2: Reliability test of the distribution model created for *Phytolacca americana* L.
